# Fréquence et déterminants de la dénutrition post-opératoire en chirurgie viscérale au Centre National Hospitalier et Universitaire Koutoucou Hubert Maga, Cotonou: Frequency and determinants of post-operative undernutrition in patients undergoing visceral surgery at the University Hospital Centre Koutoucou Hubert Maga, Cotonou

**DOI:** 10.11604/pamj.2018.29.19.10805

**Published:** 2018-01-09

**Authors:** Adébayo Cossi Alassani, Adrien Montcho Hodonou, Albert Comlan Dovonou, Gaspard Dansou Gbessi, Séraphin Ahoui, Francis Moïse Dossou, Delphin Kouassi Mêhinto

**Affiliations:** 1 Service de Médecine Interne, Centre Hospitalier Universitaire Départemental du Borgou (CHUD-B) Parakou (République du Bénin); 2 Faculté des Sciences de la Santé de l’UAC, Ecole de Nutrition, Cotonou, (République du Bénin); 3 Département de Chirurgie et Spécialités, Faculté de Médecine, Service de Chirurgie Générale du CHUD-B, Université de Parakou, Parakou (République du Bénin); 4 Département de Médecine et Spécialités, Faculté de Médecine de l’Université de Parakou, Centre Hospitalier Universitaire Département du Borgou, Parakou (République du Bénin); 5 Cliniques Universitaires de Chirurgie Viscérale, Centre National Hospitalier et Universitaire (CNHU) HKM de Cotonou (République du Bénin); 6 Département de Médecine et Spécialités, Faculté de Médecine, Université de Parakou, Unité de Néphrologie et Dialyse, Centre Hospitalier Universitaire Départemental du Borgou, Parakou (République du Bénin)

**Keywords:** Dénutrition, post-opératoire, chirurgie viscérale, Cotonou, Bénin, Undernutrition, post-operative, visceral surgery, Cotonou, Benin

## Abstract

L'objectif de cette étude était de déterminer la fréquence et les facteurs associés à la dénutrition chez les patients opérés en chirurgie viscérale en 2014. Il s'est agi d'une étude transversale, descriptive et analytique couvrant la période du 11septembre 2014 au 11 décembre 2014. La population d'étude était constituée des patients opérés en chirurgie viscérale au Centre National Hospitalier Universitaire Hubert Koutoucou MAGA de Cotonou. La dénutrition a été définie pour un indice de masse corporelle inférieur à 18,5 kg/m^2^. Au total 90 patients avaient été inclus dans l'étude, 57,78 % (52 patients) étaient de sexe masculin. La moyenne d'âge des patients était 55±6,32 ans. La dénutrition était observée chez 42 patients (46,67%). Les facteurs associés à la dénutrition chez les patients en postopératoire étaient : l'âge supérieur ou égal à 50 ans, les apports énergétique, protéique et hydrique faibles, la diarrhée, la présence de cancer, la chirurgie sur le tractus digestif, la chirurgie urgente, une perte de poids significative et l'hyperleucocytose. La dénutrition est fréquente chez les patients en postopératoire. Elle nécessite un dépistage et une prise en charge précoces.

## Abstract (en)

This study aims to determine the frequency and identify the factors associated with undernutrition among patients undergoing visceral surgery in 2014. We conducted a cross-sectional, descriptive and analytical study over the period September 11, 2014-December 11, 2014. The study population comprised patients who had undergone visceral surgery at the National University Hospital Centre Koutoucou Hubert MAGA, Cotonou. Undernutrition is defined as a body mass index less than 18.5 kg/m^2^. A total of 90 patients were included in the study, 57,78% (52 patients) were male. The average age of patients was 55±6,32 years. Undernutrition was found in 42 patients (46.67%). The factors associated with undernutrition in postoperative patients were: age greater than or equal to 50 years, low level of protein, water and energy intake, diarrhea, cancer, digestive system surgery, emergency surgery, significant weight loss and hyperleukocytosis. Undernutrition is common in postoperative patients. Hence the importance of nutritional screening and of early treatment.

## Introduction

La dénutrition qui résulte du défaut d'apport énergétique et ou protéique par rapport aux besoins de l'organisme constitue un problème majeur chez les patients hospitalisés [1]. Toutes les spécialités médico-chirurgicales sont concernées. Sa prévalence est élevée chez les patients en périopératoire. Selon Shpata et al. 40 à 50% des patients en chirurgie présentent une dénutrition à l'admission aggravée au cours de leur séjour à l'hôpital [2]. Plusieurs facteurs favorisent cette altération de l'état nutritionnel en chirurgie. Parmi ceux-ci on cite, le jeûne péripoératoire et les troubles digestifs [3]. La période postopératoire est la plus critique; le patient est soumis à la fois à une réponse inflammatoire et endocrinienne secondaire à la chirurgie, à une majoration du catabolisme et à une anorexie dont l'intensité et la durée sont proportionnelles à la sévérité de l'acte chirurgical [4]. L'évaluation de l'état nutritionnel des patients pendant cette période s'avère indispensable en vue d'une prise en charge adéquate dans la mesure où, la dénutrition a un impact négatif sur le patient en chirurgie. Elle augmente le risque des complications postopératoires. Les patients dénutris ont une morbidité et une mortalité postopératoires plus élevées [5,6]. La dénutrition favorise une dépression de l'immunité, un risque accru de développer les infections, une inefficacité du traitement médicamenteux, un retard de cicatrisation des lésions postopératoires et une augmentation de la durée d'hospitalisation [7,8]. Une perte de poids de plus de 20%, facteur de risque de la dénutrition est connue depuis 1936 comme associée à une augmentation de la mortalité chez les patients opérés pour un ulcère duodénal [7]. Un bon état nutritionnel réduit la morbi-mortalité postopératoire [3,9]. Contrairement aux pays développés, l'appréciation de l'état nutritionnel des patients hospitalisés est loin d'être une priorité dans les pays en voie de développement notamment au Bénin. La présente étude s'est intéressée à la dénutrition en postopératoire afin de déterminer sa fréquence et ses facteurs associés.

## Méthodes

**Type et période d'étude:** Il s'est agi d'une étude observationnelle, transversale, descriptive et analytique réalisée du 11septembre au 11 décembre 2014.

**Population d'étude:** Elle était constituée des patients en postopératoire suivis dans le service de chirurgie viscérale du Centre National Hospitalier Universitaire Hubert Koutoucou Maga de Cotonou constitué de deux cliniques universitaires de chirurgie viscérale.

**Critères d'inclusion:** Les malades opérés d'une affection dans la période d'étude en urgence ou en chirurgie programmée hospitalisés ayant fait au moins dix jours d'hospitalisation étaient inclus dans notre étude. Critères d'exclusion : les malades opérés décédés avant sept jours, et ceux en coma ou en réanimation chez qui les données ne peuvent pas être prises étaient exclues.

**Critères de non inclusion:** Les autres malades non opérés hospitalisés dans le service.

**Définition de la dénutrition (variable dépendante):** La dénutrition a été définie par l'indice de masse corporelle, ainsi tout patient ayant un indice de masse corporelle inférieur à 18,5 kg/m^2^ était considéré comme dénutri ou par une perte de poids significative supérieure ou égale à 2 % du poids habituel en une semaine. Les autres variables étudiées étaient: sociodémographiques ; le niveau socio-économique, considéré comme bon lorsque les patients étaient en mesure d'honorer sans difficultés les prescriptions médicales. Les antécédents de diabète défini par une glycémie à jeun supérieure ou égale à 1,26 g/L à deux reprises séparées de 24 heures l'une de l'autre ou les sujets connus et déjà traités comme tel; l'anémie définie par un taux d'hémoglobine inférieur à 12 g/L chez les hommes et 11 g/L chez les femmes; l'hyperleucocytose était définie pour un taux sanguin de leucocytes supérieur à 8000/ml. L'apport énergétique a été évalué par le journal alimentaire sur 72 heures. L'apport énergétique considéré était celui de la moyenne de trois jours successifs. Les besoins énergétique et protéique normaux considérés étaient respectivement 35 kcal/kg/jour et 20% de l'apport énergétique. Les besoins énergétiques ont été multipliés par 1,2 pour prendre en compte le facteur de stress postopératoire. Les patients dont l'apport énergétique était inférieur au besoin de plus de 200 kcal avaient un apport énergétique faible. Ceux dont l'apport énergétique était supérieur au besoin de plus 200 kcal avaient un apport énergétique élevé. Les patients dont l'apport protéique était inférieur au besoin de plus de 10 g ont un apport protéique faible. Ceux dont l'apport protéique était supérieur au besoin de plus 10 g ont un apport protéique élevé. L'apport en eau était calculé en faisant la somme de l'eau de boisson, de l'eau contenue dans les aliments liquides et des solutés perfusés. Les besoins en eau étaient de 1 mL pour 1 kcal c´est-à-dire autant de kilocalorie autant de mL d'eau. En cas de fièvre, les besoins hydriques étaient augmentés avec un supplément de 200 mL pour un degré de température au dessus de 38 degrés. Les patients dont l'apport hydrique était inférieur au besoin de plus de 500 mL avaient un apport hydrique faible. Ceux dont l'apport hydrique était supérieur au besoin de plus 500 mL avaient un apport hydrique élevé. En dehors des limites définies, l'apport en énergie, en protéine ou en eau était considéré comme normal. La chirurgie était qualifiée de digestive lorsque qu'elle intéressait le tube digestif. Les interventions chirurgicales qui n'altéraient pas l'intégrité fonctionnelle du tube digestif comme l'appendicectomie, des cures herniaires non étranglées, l'ablation des bourrelets hémorroïdaires n'étaient pas inclues dans les chirurgies digestives.

**Analyses des données:** Le logiciel Epi Info 7 avait été utilisé pour l'analyse des données. Le test de khi carré a été calculé et une p value inférieure à 0,05 a été considérée comme significative. Pour étudier la force de l'association, les facteurs associés significatifs ont été soumis à la régression logistique avec un intervalle de confiance de 95%.

**Considérations éthiques:** Notre étude purement observationnelle, n'a pas eu besoin de l'avis d'un comité d'éthique. Toutefois, l'autorisation des chefs de service avait été obtenue de même que les malades avaient donné leur consentement. La confidentialité des données recueillies avait été garantie par le système d'anonymat.

## Résultats

Parmi les 90 patients inclus dans l'étude, 57,78 % (52 patients) étaient de sexe masculin. La moyenne d'âge des patients était 55±6,32 ans. Les patients ayant un bon niveau socioéconomique représentaient 57,78 % (52 patients) de la population d'étude. Les deux tiers des patients avaient un apport énergétique insuffisant, six patients sur 10 (62,22%) avaient un apport protéique insuffisant. Les trois quarts des patients (75,66%) avaient un apport en eau normal. Les troubles digestifs observés étaient la nausée chez 34 patients (37,78 %), les vomissements chez 14 patients (15,56%), la sensation de satiété précoce chez 54 patients (60%), la diarrhée chez 6 patients (6,67%). La douleur était ressentie par près de 8 patients sur 10 (77,78%), la dysphagie était observée chez 10 patients (11,11%). La dyspnée était observée chez 6 patients (7,69%). Le diabète sucré était présent chez 14 patients (15,56%) tandis que 10 patients (11,11%) étaient porteurs de cancer. Près du quart des patients (22,22 %) avaient un antécédent de chirurgie digestive. La chirurgie digestive était réalisée chez 80 patients (88,89%). Dans le tiers des cas, il s'est agi d'une chirurgie réalisée en urgence. L'hyperleucocytose était observée chez 58 patients (64%) Tableau 1. L'état nutritionnel des patients était caractérisé par la dénutrition observée chez 42 patients (46,67%), une perte de poids significative chez deux tiers des patients et l'anémie chez 74 patients (82,22%) Tableau 2. Les facteurs associés à la dénutrition chez les patients en postopératoire en analyse univariée étaient : l'âge supérieur ou égal à 50 ans, les apports énergétique, protéique et hydrique faibles, les troubles digestifs tels que la nausée, les vomissements, et la diarrhée, la dyspnée, le diabète sucré, la présence de cancer, l'antécédent de chirurgie digestive, la chirurgie digestive, la chirurgie urgente, une perte de poids significative, l'anémie et l'hyperleucocytose (Tableau 3). En analyse multivariée, les facteurs associés à la dénutrition chez les patients en postopératoire étaient : l'âge supérieur ou égal à 50 ans, les apports énergétique, protéique et hydrique faibles, la diarrhée, la présence de cancer, la chirurgie digestive, la chirurgie urgente, une perte de poids significative et l'hyperleucocytose (Tableau 4 ).

**Tableau 1:** Caractéristiques générales de la population d’étude (n=90)

Variables	n (%)	Variables	n (%)
**Sexe**		**Douleur**	
Féminin	38(42,22)	Présente	70(77,78)
Masculin	52(57,78)	Absente	20(22,22)
**Age**		**Dysphagie**	
<50 ans	46(51,11)	Présente	10(11,11)
≥50 ans	44(48,89)	Absente	80(88,89
**Niveau socioéconomique**		**Dyspnée**	
Mauvais	38(42,22)	Présente	6(7,69)
Bon	52(57,78)	Absente	84(92,31)
**Apport énergétique**		**Antécédent de diabète**	
Bas	60(66,67)	Présent	14(15,56)
Normal	30(33,33)	Absent	76(84,44)
**Apport protéique**		**Antécédent de cancer**	
Bas	56(62,22)	Présent	10(11,11)
Normal	34(37,78)	Absent	70(88,89)
**Apport en eau**		**Antécédent de chirurgie digestive**	
Bas	22(24,44)	Présent	20(22,22)
Normal	68(75,66)	Absent	70(77,78)
**Nausées**		**Chirurgie digestive**	
Présentes	34(37,78)	Oui	80(88,89)
Absentes	56(62,22)	Non	10(11,11)
**Vomissements**		**Modalité de chirurgie**	
Présents	14(15,56)	Programmée	30(33,33)
Absents	76(84,44)	Urgente	60(66,67)
**Satiété précoce**		**Taux de leucocytes**	
Présente	54(60)	Elevé	58(64)
Absente	36(40)	Normal	32(36)
**Diarrhée**			
Présente	6(6,67)	-	-
Absente	84(93,33)	-	-

**Tableau 2:** Etat nutritionnel des patients en postopératoire

Variables	n	%
**Indice de masse corporelle (IMC en kg/m^2^)**		
IMC < 18,5	42	46,67
18,5 ≤ IMC < 25	30	33,33
25 ≤ IMC < 30	13	14,44
IMC ≥ 30	5	5,56
**Perte de poids**		
< 2%	30	33,33
≥ 2%	60	66,67
**Anémie**		
Présente	74	82,22
Absente	16	17,73

**Tableau 3:** Facteurs associés à la dénutrition chez les patients en postopératoire (Analyse multivariée)

Variables	Odds Ratio (IC à95%)	p-value
**Age**		0,0000
<50 ans	1	
≥50 ans	3,77 (2,25; 5,18)	
**Apport énergétique**		0,0000
Bas	1	
Normal	0,4 (0,26; 0,75)	
**Apport en protéine**		0,021
Bas	1	
Normal	0,33(0,17; 0,84)	
**Apport en eau**		0,0000
Bas	1	
Normal	0,23 (0,12; 0,39)	
**Nausées**		0,345
Présentes	1	
Absentes	0,8 (0,7; 1,56)	
**Vomissement**		0,137
Présents	1	
Absents	0,75 (0,56; 1,23)	
**Diarrhée**		0,026
Présente	1	
Absente	0,58 (0,31; 0,79)	
**Perte de poids significative**		0,0000
Oui	1	
Non	0,30(0,19; 0,63)	
**Dyspnée**		0,23
Présente	1	
Absente	0,91 (0,77; 1,12)	
**Antécédents de diabète**		0,572
Présent	1	
Absent	0,88 (0,79; 1,23)	
**Antécédents de cancer**		0,0000
Présent	1	
Absent	0,20 (0,17; 0,78)	
**Antécédents de chirurgie digestive**		0,356
Présent	1	
Absent	0,86 (0,70; 1,17)	
**Chirurgie digestive**		0,046
Oui	1	
Non	0,28 (0,58; 0,78)	
**Modalité de chirurgie**		0,0000
Programmée	1	
Urgente	2,77 (1,63; 5,12)	
**Taux de leucocytes**		0,00056
Elevé	1	
Normal	0,52(0,14; 0,77)	
**Anémie**		0,452
Présente	1	0,452
Absente	0,82(0,71; 1,31)	

**Tableau 4:** Facteurs associés à la dénutrition chez les patients en postopératoire (Analyse univariée)

Variables	n (N)	p	Variables	n (N)	p
**Sexe**		0,458	**Douleur**		0,7
Féminin	16 (22)		Présente	32 (38)	
Masculin	26 (26)		Absente	10 (10)	
**Age**		0,00000	**Dysphagie**		0,6
< 50 ans	5 (41)		Présente	4 (6)	
≥ 50 ans	37 (7)		Absente	38 (42)	
**Niveau socioéconomique**		0,458	**Dyspnée**		0,01
Mauvais	26 (26)		Présente	6 (0)	
Bon	16 (22)		Absente	42 (42)	
**Apport énergétique**		0,00000	**Antécédent de diabète**		0,00001
Bas	38 (22)		Présent	14 (0)	
Normal	4 (26)		Absent	28 (48)	
**Apport protéique**		0,001	**Antécédent de cancer**		0,00033
Bas	42 (38)		Présent	10 (0)	
Normal	10 (00)		Absent	32 (48)	
**Apport en eau**		0,00000	**Antécédent de chirurgie digestive**		0,0002
Bas	22 (0)		Présent	16 (4)	
Normal	18 (50)		Absent	24 (46)	
**Nausées**		0,007	**Chirurgie digestive**		0,0017
Présentes	22 (12)		Oui	42 (38)	
Absentes	20 (36)		Non	0 (10)	
**Vomissements**		0,04	**Modalité de chirurgie**		0,0017
Présents	10 (14)		Programmée	7 (23)	
Absents	32 (48)		Urgente	35 (25)	
**Satiété précoce**		0,6	**Taux de leucocytes**		0,002
Présente	24 (30)		Elevé	34 (24)	
Absente	18 (18)		Normal	8 (24)	
**Diarrhée**		0,006	**Anémie**		0,0135
Présente	6 (0)		Présente	39 (35)	
Absente	36 (48)		Absente	3 (13)	
**Perte de poids significative**		0,00000			
Oui	28 (2)				
Non	14 (46)				

**N** = nombre de patients dénutris, **N** = nombre de patients non dénutris

## Discussion

Les complications postopératoires sont multiples et ont plusieurs origines. Elles peuvent être relatives à la pathologie en cause, à l'intervention chirurgicale mais aussi à l'état nutritionnel périopératoire du patient. L'influence de l'état nutritionnel des patients sur la morbidité et la mortalité postopératoire est prouvée ; les patients dénutris ont significativement une mortalité postopératoire plus élevée [10,11]. La connaissance de l'ampleur et des facteurs associés à la dénutrition sont d'une importance capitale. Dans la présente étude, l'appréciation de l'état nutritionnel des patients s'est intéressée au dépistage de la dénutrition. Elle a été faite par l'indice de masse corporelle qui est un outil reconnu par sa simplicité et sa fiabilité. Le dosage de l'albuminémie et celui de la préalbuminémie pourraient aider à mieux apprécier la dénutrition. Dans le contexte post opératoire, la synthèse de l'albumine ou de la pré albumine est diminuée au profit des protéines inflammatoires. L'appréciation de l'état nutritionnel anthropométrique semble alors plus adaptée. Les résultats de l'étude montre une fréquence élevée de la dénutrition chez les patients en postopératoire. Près de la moitié des patients (46,67%) étaient dénutris. La dénutrition en postopératoire a été également la préoccupation de plusieurs auteurs. Shim et al. [5] et Beaton et al [12] ont rapporté respectivement une fréquence de dénutrition post-opératoire de 26,3% et 16%. La fréquence élevée de la dénutrition dans la présente étude s'explique par le peu d'attention qui est accordée à l'état nutritionnel des patients hospitalisés. Il est donc indispensable de faire un dépistage précoce de la dénutrition et une prise en charge adéquate chez les patients en périopératoire. En chirurgie digestive, du fait de la mise au repos du tube digestif et des troubles digestifs, la prévalence de la dénutrition est très élevée et peut atteindre 84,9% [2].

Les facteurs associés à la dénutrition retrouvés dans la présente étude sont en analyse multivariée étaient: l'âge de 50 ans et plus, les apports énergétique, protéique et hydrique faibles, la diarrhée, la présence de cancer, la chirurgie réalisée en urgence, une perte de poids significative et l'hyperleucocytose. Plusieurs études ont retrouvé les mêmes associations avec la dénutrition. C'est le cas des études de Chambrier et al et Zhou et al. Pour ce qui est de l'association entre l'âge élevé et la dénutrition [4,13]. Pour assurer un bon état nutritionnel, un apport adéquat en nutriments et une bonne absorption de ceux-ci s'avèrent nécessaire. L'absence de l'intégrité du tube digestif et un apport insuffisant en nutriment peuvent être sources de dénutrition. Ceci explique l'association entre les troubles digestifs tels que la diarrhée et les apports énergétiques faibles en énergie et en protéine avec la dénutrition. L'eau est le principal constituant de l'organisme. Le maintien de l'équilibre hydrique est sujet à un apport adéquat en eau et une bonne absorption. Un apport faible en eau peut favoriser la déshydratation voire la dénutrition même si les apports en énergie et en protéine sont normaux. L'association entre l'apport énergétique faible et la dénutrition est aussi observée dans les études de Gheorghe et al et de Jeejeebhoy et al. [14,15]. Pour Khamnuan et al, le faible apport en protéine favorise non seulement la dénutrition mais aussi l'hypoprotidémie qui est associée à un sepsis [16]. L'effet négatif de l'apport faible en énergie et de la perte de poids sur la mortalité postopératoire des patients a été mis en exergue dans l'étude de Kwag et al [17]. Dans les études de Chambrier et al. et de Calon et al. l'association entre les troubles digestifs et la dénutrition a été également retrouvée. Leandro-Merhi et al. se sont intéressés à l'association entre la présence de cancer et la dénutrition en chirurgie. Les résultats de leur étude montrent que le cancer est associé à la dénutrition en chirurgie [18]. Durakba'a et al. ont rapporté l'association entre la chirurgie non programmée et la dénutrition [19] tandis que Zhou et al. ont noté que la chirurgie digestive est un facteur de risque de dénutrition [13]. de Luis et al. ont retrouvé une association semblable à la nôtre pour ce qui est de l'association dénutrition et perte de poids [7]. L'hyperleucocytose peut être le reflet d'une infection. L'infection étant un facteur favorisant la dénutrition. Il se constitue donc un cercle vicieux préjudiciable au pronostic des patients. L'association entre l'hyperleucocytose et la dénutrition ne fait pas l'unanimité. Fernández et al n'ont retrouvé aucune association entre le taux sanguin de leucocytes et la dénutrition [20].

## Conclusion

La dénutrition est fréquente chez les patients en postopératoire. Vu l'impact négatif de la dénutrition sur la mortalité et la morbidité postopératoires des patients, son dépistage et sa prise en charge s'avèrent indispensables. La formation du personnel de santé sur les notions de nutrition ou le recrutement des professionnels de la nutrition sont impératifs.

### Etat des connaissances actuelles sur le sujet

- La dénutrition post opératoire aggrave la morbimortalité;
- Fréquence varie;
- Ces facteurs peuvent être maitrisés.

### Contribution de notre étude à la connaissance

- La dénutrition post-opératoire est ignorée dans notre contexte, d'où la forte fréquence, presque un malade sur deux;
- L'apport protéino-calorique est quasi-déficitaire chez deux patients sur 3 et l'apport en eau est insuffisant chez trois patients sur trois;
- Aucune prévention n'est réalisée dans notre contexte.

## Conflits d’intérêts

Les auteurs ne déclarent aucun conflit d'intérêts.
